# Knowledge, sources and use of family planning methods among women aged 15-49 years in Uganda: a cross-sectional study

**DOI:** 10.11604/pamj.2016.24.39.5836

**Published:** 2016-05-10

**Authors:** Stephen Galla Alege, Joseph KB Matovu, Simon Ssensalire, Elizabeth Nabiwemba

**Affiliations:** 1 Makerere University School of Public Health-CDC Fellowship Program Kampala, Uganda; 2 Uganda Health Marketing Group, Kampala, Uganda; 3 Makerere University School of Public Health, Kampala, Uganda; 4 Program for accessible Health Communication and Education, Kampala, Uganda

**Keywords:** Knowledge, use, family, planning, methods, women, Uganda

## Abstract

**Introduction:**

Lack of knowledge of where to obtain correct family planning (FP) information and methods can be a critical barrier to eventual uptake of FP services. We assessed knowledge, sources and use of FP methods among women of reproductive age in rural Uganda.

**Methods:**

This secondary analysis uses data from a larger cross-sectional study conducted to measure changes in perceptions towards long-term and reversible contraceptive use among 2,033 women of reproductive age (15-49years) resident in 34 districts of Uganda. Both users and non-users of FP methods were interviewed. Data were analyzed using STATA statistical software, version 12.

**Results:**

Majority of the women were less than 30 years of age (64.3%). Nearly three-quarters were married (73.1%), 51.1% had primary education and more than half (57%) were engaged in employment. Knowledge of FP methods was universal (98.1%). Clinic providers (60.4%), friends (56.9%) and the media (51.3%) were the most trusted sources of contraceptive information. Government (27.6%) and private (21.1%) health facilities were the main sources of modern FP methods. Sixty two per cent of women reported current use of any FP method. Among non-users of FP, injectables (50.4%), implants (22.8%) and pills (20.2%) were the most preferred FP methods.

**Conclusion:**

Our findings show that knowledge of FP methods is almost universal and that six in ten women use any FP method. Clinic providers, friends and the media are the most trusted sources of FP information. Government and private health facilities are the main sources of FP services.

## Introduction

Use of family planning (FP) methods can contribute to a substantial reduction in fertility and reduce the proportion of unwanted pregnancies as well as maternal deaths that would otherwise occur in the absence of contraception. In 2008, contraceptive use averted over 250,000 maternal deaths worldwide by reducing unintended pregnancies, which is equivalent to 40% of the 355,000 maternal deaths that occurred in that year [1]. However, while knowledge of FP has significantly increased over the last decade; uptake of FP services has remained low, especially in sub-Saharan Africa. Total fertility rates in sub-Saharan Africa remain critically high with an average of 5.5 children per woman of reproductive age in most African countries [2]. Although efforts to expand and promote the use of family planning services in sub-Saharan Africa have received recent international attention, the unmet need for modern family planning methods continues to remain high [3, 4]. Fear of contraceptive side effects and associated treatment costs, cultural barriers and low male involvement continue to hamper effective use of FP services in most countries [5]. Lack of knowledge of where to obtain FP methods [6] and lack of information on what women consider to be trusted sources of FP information and services, are key barriers that affect access to and utilization of FP methods in most sub-Saharan African countries [6, 7]. This calls for interventions that can simultaneously improve women′s knowledge of FP methods and improve uptake of such services by utilizing sources that they trust most. However, despite prior research on the uptake of contraceptive services in sub-Saharan Africa [8–10], there is still limited research on the extent to which women are aware of specific methods of family planning; and if they do, where they obtain this information from. There is virtually no literature on what women consider to be the most trusted sources of FP information and services, yet knowledge of such sources is important for targeted FP promotion [11]. This presents a missed opportunity for full-scale expansion of FP programs and calls for a need to document and understand not only whether women have correct knowledge of FP methods but also whether they know where to obtain the methods from, and whether they trust those sources.

In Uganda, uptake of contraceptive services continues to remain low, with only 26% of married women currently using FP methods [5]. Although there have been method-specific increases in the use of short-term methods, uptake of long-term and permanent methods remains low over the years. For instance, while uptake of contraceptive pills has increased from 2.3% in 2006 to 2.9% in 2011 and injectables from 7.7% in 2006 to 14.1% in 2011; uptake of intra-uterine device (IUD) has increased only slightly from 0.1% in 2006 to 0.5% in 2011while uptake of female sterilization has only increased slightly from 1.7% in 2006 to 2.9% in 2011 [5, 12]. Awareness of specific FP methods also follows a similar trend, with high knowledge reported for short- but not long-term methods [13]. While FP knowledge is almost universal in Uganda, only 18.5% of expectant mothers receive FP information from health facilities during prenatal care clinics at public health facilities and 58.1% of expectant mothers discuss family planning with their spouses [14]. To guide the implementation of a program for improved uptake of long-term and permanent FP methods by Program for Accessible Health Communication and Education (PACE) in Uganda, a study was conducted to document the current levels of knowledge of FP methods, the sources where women obtain FP information and services, which sources of FP information that women trust, and current/preferred use of FP services. PACE is a local nongovernmental organization with a mission to improve the health of Ugandans through social marketing. One of its objectives is to increase access to and utilization of long-term FP methods among women of reproductive age in Uganda.

## Methods

### Study site and population

This study uses data obtained from a larger study on Measuring Change in Perceptions towards Long-Term and Reversible Contraceptives (LARCs) Use among Women of Reproductive Age in Uganda that was conducted between July and September 2012. Details about the larger study have been reported elsewhere [15]. Briefly, the larger study was conducted in 34 districts that were selected from five a priori demarcated regions (Central, Eastern, South-west, Western and Northern) that were used for the 2011 Uganda Demographic and Health Survey [5]. Eight districts were selected from the Central region, ten from the Eastern region, three from the South-western region, six from the Western region and seven from the Northern region. Within each region, only those districts in which PACE was implementing the above-mentioned FP promotional campaign were selected for the survey. The study population was composed of women of reproductive age (15-49 years), resident in the selected districts. Within each district, all women residing within the catchment area (i.e. sub-County) of a ProFam-branded health facility at the time of the survey were eligible for participation irrespective of whether they had ever obtained FP services from the ProFam-branded health facility or not. ProFam is a social franchise (franchised by PACE) of private health clinics providing a range of quality health services within the study districts. ProFam-branded health facilities offer FP services including long-term and reversible contraceptives (LARCs), pills and injectables.

### Data collection procedures

Data were collected using structured, interviewer-administered questionnaires. Data were collected on: knowledge of FP, sources of FP methods, sources of FP information, trusted sources of FP information; current, preferred and intended use of FP methods. Knowledge of FP methods was assessed by asking respondents whether or not they knew or had ever heard about any FP methods. Respondents were asked about sources of FP information and methods, as well as their levels of trust of the sources of FP information cited. With regard to trusted sources of information, respondents were asked to identify and rank (in descending order) three most trusted sources of FP information. Use of FP methods was assessed by asking married women about whether or not they were currently using any FP methods. Current users of FP methods were asked about the sources of FP supplies. Non-users of FP methods were asked about their most preferred methods while both users and non-users were asked about which FP methods they intended to use in the next twelve months.

### Measures

To assess knowledge of FP methods, women were asked if they had ever heard about methods that can be used to avoid pregnancies. Those that responded in the affirmative were further asked about what FP methods they knew or had heard of. Women were considered knowledgeable about FP methods if they (spontaneously or after prompting) mentioned at least one FP method, regardless of whether it was a traditional or modern method. To improve clarity about the different methods, descriptive summaries of each method were provided in the questionnaires to help interviewers in explaining specific methods if the respondent did not know them.*Source of family planning information* was defined as any place or area or person from whom a woman obtained information about any method of family planning while *source of family planning method* was defined as any place, area or person from whom a woman obtained family planning methods, regardless of whether these were traditional or modern methods. Trusted sources of FP information were defined as self-reported sources of FP information that women spontaneously mentioned as their most trusted sources of FP information. Use of family planning methods was divided into three categories: current use, preferred use and intention to use. Current use was assessed by asking women if they were “currently doing something to avoid getting pregnant” and those that responded in the affirmative were asked: *“What is the main method you are currently using to avoid getting pregnant?”* Preferred use was assessed among non-users and was defined as self-reported preference for a family planning method by women who were not currently using any method of family planning. Intention to use FP methods was defined as a self-reported plan to use a family planning method in the next twelve months by both users and non-users.

### Data Analysis

Data were entered into SPSS and transferred into STATA at the time of analysis. We computed descriptive statistics on the socio-demographic characteristics of the study respondents (i.e. age, marital status, level of education, religious affiliation, etc.), knowledge of the various FP methods (i.e. short- or long-term and traditional or modern methods), sources of FP information and methods, trusted sources of FP information; and current, preferred, and intended use of FP methods. Data are presented in form of tables, frequencies and percentages.

### Ethical consideration

Ethical approval for the bigger study was obtained from the Mildmay Uganda Research &Ethics Committee. Informed consent was sought from subjects involved in the data collection. Further, confidentiality of collected information was insured by clients not providing names or any other identifiers. Respondents were made comfortable and protected from any form of harm during the course of data collection.

## Results

### Respondents’ characteristics

A total of 2,033 women aged 15-49 years were enrolled into this study. Majority of these were young (below 30 years), with nearly half (48.8%) aged 20-29 years (Table 1). Majority of the respondents were currently married (73.1%). Nearly all the respondents had some level of formal education (93.3%); half (51.1%) of the respondents had primary education. Majority of the respondents were Christians (77.7%). Most of the respondents work to earn an income (57.6%); however, 42.4% reported that they were not engaged in any form of paid work. Majority of those who work derive their income from business (40.8%) and farming (33.3%). Majority of the respondents cited their spouse as the primary household provider (45.1%) while 20.9% reported that they and their husbands were the primary household providers (Table 1).

**Table 1:** Population characteristics

Characteristics	Number(N = 2,033)	Percentage (%)
**Age Group**		
15-19	316	15.5
20-24THE GOOD WIFE	533	26.2
25-29	459	22.6
30-34	321	15.8
35-39	204	10.0
40-44	118	5.8
45-49	82	4.0
**Marital Status**		
Single	340	16.7
Widow/divorced/separated	207	10.2
Currently married	1,486	73.1
**Level of education**		
None/no formal education	118	5.8
Primary	1,038	51.1
Secondary or higher	877	43.1
**Religious Affiliation**		
None	45	2.2
Muslim	409	20.1
Catholic	703	34.6
Protestant	601	29.6
Other Christian	275	13.5
**Works to earn an income**		
No	863	42.4
Yes	1,170	57.6
Income Source (N=1,170)*		
Informal	71	6.0
Farming	390	33.3
Private Sector	76	6.5
Civil Service	50	4.3
Casual work	78	6.7
Business	477	40.8
Clothes vendor/pensioner	28	2.4
**Primary household provider**		
Spouse	917	45.1
Self	261	12.8
Both hubby and self	424	20.9
Parents	326	16.0
Other relatives	105	5.2

* Expressed out of those who reported working to earn an income

### Knowledge of FP methods among women of reproductive age

Knowledge of FP methods was nearly universal (98.1%). Method-specific knowledge was highest for short-term methods (e.g. male condoms (98.3%), pills (97.9%) and injectables (97.6%)). Knowledge of long-term FP methods (implants (91.7%); intra-uterine devices (89.1)) was equally high as was knowledge of permanent methods (female (79.3%); male sterilization (77.6%)). However, knowledge of lactational amenorrhea and emergency contraceptives was the lowest at 71.9% and 40.1% respectively (Table 2).

**Table 2:** Knowledge of family planning methods

Methods	Number (N = 2,033)	Percentage (%)
Knowledge of any FP method	1,995	98.1
Knowledge of specific methods*		
Male condoms	1,999	98.3
Pills	1,991	97.9
Injectables	1984	97.6
Implants	1,865	91.7
Withdrawal	1,827	89.9
IUD	1,812	89.1
Female condoms	1,737	85.4
Periodic abstinence	1,686	82.9
Female sterilization	1,612	79.3
Male sterilization	1,577	77.6
Lactational amenorrhea	1,462	71.9
Emergency contraceptives	815	40.1
Other traditional method	283	13.9

* Knowledge of each method was assessed independently out of 100%

### Trusted sources of family planning information and methods

When respondents were asked to mention any three sources of FP information that they trusted, 60.4% (n = 2,033) indicated that they trusted clinic providers, over half (56.9%) mentioned friends while half (51.3%) mentioned the media. Only 33% mentioned health education as one of the three trusted sources of FP information (Table 3). Of women who were currently using a family planning method (n = 1,265), 412 (32.6%) reported that they were using natural/traditional family planning methods (i.e. withdrawal, periodic abstinence, lactational amenorrhea or any other traditional methods) while 67.4% were using a modern method. Of those using a modern FP method (853), 40.9% reported obtaining the methods from a government health facility, 31.3% from a private health facility and 7.1% from a mission hospital/clinic. Interestingly, only 2.2% of current users of FP methods reported obtaining the methods from friends (Table 4).

**Table 3:** Most trusted sources of family planning information*

Source		Number (N = 2,033)	Percentage (%)
Clinic providers		1,229	60.4
Friends		1,156	56.9
Media		1,043	51.3
Health education		671	33.0
Family		345	17.0
Partners		310	15.2
Community health worker		233	11.5
School		166	8.2
Road/drama shows		71	3.5
ProFam clinics		28	1.4
Beauty salons		10	0.5
Other sources		161	7.9

* Each source was assessed independently out of 100%

**Table 4:** Sources of family planning services among current users of FP methods

Method	Number (N = 1,265)	Percentage (%)
Natural/traditional method*	412	32.6
Modern methods**	853	
Government health facility	349	40.9
Private health facility	267	31.3
Mission hospital/clinic	61	7.1
Drugstore	49	5.7
Profam clinic	31	3.6
Pharmacy	29	3.4
Friends	19	2.2
Other sources	48	5.6

* Traditional/natural methods include lactational amenorrhea, withdrawal and periodic abstinence

** Modern methods include male condoms pills, injectables, implants, IUD, female condoms, female sterilization and male sterilization

### Current and preferred use of family planning methods

Table 5 shows current use family planning methods among women of reproductive age. Overall, 62.2% (1,265 of 2,033) of women reported that they were currently using a family planning method; 76.3% of these were using a modern method. Current use of FP methods was highest for injectables (33%), lactational amenorrhea (16.7%), female sterilization (12.3%) and male condoms (11%). Current use of IUD (7.2%) was low as was use of pills (6.7%). Women who were not currently using any method of FP (n = 768) were asked about what method of FP they would prefer if they were to use any FP methods. Half of the women reported that they preferred injectables (50.4%), one-quarter preferred implants (22.8%) and pills (20.2%), 15.7% preferred male condoms (15.7%) while 10.8% preferred to use IUDs. Only 6.1% of women preferred to use female sterilization as a method of family planning while female condoms were preferred by only 4.2% (Table 6).

**Table 5:** Current use of family planning methods

Method	Number (N = 1,265)	Percentage (%)
Current use of any FP method	1,265	62.2
Current use of modern FP methods*	965	76.3
Method-specific use		
Injectables	417	33.0
Lactational amenorrhea	212	16.7
Female sterilization	156	12.3
Male condoms	139	11.0
Intra-uterine device	91	7.2
Pills	85	6.7
Periodic abstinence	55	4.3
Male sterilization	48	3.9
Foams/gel	24	1.9
Withdrawal	21	1.7
Other modern FP methods	05	0.4
Other traditional FP methods	12	0.9

* Expressed out of those who reported currently using any FP method

**Table 6:** Preferred family planning methods among women who were not using a family planning method

Method	N = 768	Percentage (%)*
Injectables	387	50.4
Implants	175	22.8
Pills	155	20.2
Male condoms	121	15.7
Intra-uterine device	83	10.8
Periodic abstinence	56	7.3
Female sterilization	47	6.1
Female condoms	32	4.2
Withdrawal	27	3.5
Lactational amenorrhea	16	2.1
Other methods	22	2.9

* Percentages expressed independently for each FP method

### Intended use of family planning methods

Among current users (n = 1,265), 35.1% intended to use injectables, 18.2% intended to use implants, 8.5% intended to use pills while 6.3% intended to use male condoms. Among non-users (n = 768), 20.8% intended to use injectables, 8.9% intended to use implants, 4.6% intended to use female sterilization, while 3.3% intended to use pills and male condoms. It is important to note that slightly more than half of non-users (53.5%) did not intend to use any method of family planning in the next twelve months (Table 7).

**Table 7:** Intention to use family planning methods among both users and non-users of family planning in the next twelve months

Method	Current FP Users		Non-FP Users	
	N = 1,265	%	N = 768	%
Injectables	444	35.1	160	20.8
Implants	230	18.2	68	8.9
Pills	108	8.5	25	3.3
Male condoms	80	6.3	25	3.3
Periodic abstinence	66	5.2	13	1.7
Female sterilization	61	4.8	35	4.6
Intra-uterine device	52	4.1	11	1.4
Withdrawal	23	1.8	04	0.5
Lactational amenorrhea	12	0.9	04	0.5
Female condoms	05	0.4	00	0.0
Emergency contraception	04	0.3	01	0.1
Male sterilization	01	0.1	00	0.0
Other method/unsure	179	14.2	11	1.4
No intention to use FP	-	-	411	53.5

## Discussion

Our study of knowledge, sources and use of FP methods among women aged 15-49 years in Uganda shows that knowledge of FP methods was almost universal and that six in every ten women reported currently using a method of family planning. We found that clinic providers, friends and the media were the most trusted sources of contraceptive information while government and private health facilities were the main sources of FP methods. There was a high preference for and intention to use short-term methods (e.g. injectables) among both users and non-users of FP methods. However, although preference for permanent methods (i.e. female sterilization) was low, preference for and intention to use long-term methods (e.g. implants) came second to injectables among both users and non-users. This is likely to have been a result of the FP promotional campaign that is being implemented by PACE in the study districts. It is important to note that more than half of non-users did not intend to use any FP methods in the next 12 months, suggesting a need for innovative and more targeted approaches to reach non-users. The finding that clinic providers, friends and the media were the most trusted sources of contraceptive information is consistent across studies [16–19] and suggests a need to expand the spectrum of communication channels - beyond formal, clinical-based approaches - to include informal mechanisms (e.g. friends) and the media to disseminate information on contraceptive products and services particularly to non-users. A study conducted among women aged 15-49 years in rural southern Nigeria found that majority of the respondents (40.6%) got their information through friends [20] while another study conducted in Pakistan found that television (26%) and relatives (24%) were the leading sources of family planning information [18]. In Guinea, Howard et al [19] found that reproductive health groups were the leading sources of information on family planning methods. Collectively, these findings suggest a need for targeting informal mechanisms and the media as alternative approaches for promoting FP use especially among non-users. We found that government health facilities were the leading sources of family planning methods. This is not surprising considering that these facilities tend to provide services free of charge thus removing one of the key barriers to accessing FP services [21, 22]. However, our findings also show that private health facilities are a good source of FP methods, suggesting a need to equip them with supplies so that women who prefer to obtain their supplies from private facilities are not deprived of these methods. In addition, the fact that private facilities came second to government facilities suggests a need for strong public-private partnerships to increase access to and utilization of FP methods among women of reproductive age in Uganda.

Our study has got a number of limitations. In the first place, this study was conducted in districts where there was an ongoing FP promotional campaign, and thus, the high level of uptake of FP methods reported could be attributed to this campaign rather than a general trend among Ugandan women. We found that the uptake of and preference for long-term and permanent methods was equally higher than has been reported in other studies [23, 24], suggesting that these findings should be interpreted with the promotional campaign in mind. However, there is evidence to show that uptake of long-term and permanent methods is increasing in Uganda [25] and that the preference for long-term and permanent methods as depicted through this study could be a true reflection of women's desires for long-term and permanent contraception. This is an area that warrants further research to explore preference for long-term and permanent methods in other Ugandan districts that do not have any ongoing FP promotional campaigns. The other limitation is that this study did not explore several aspects pertaining to FP use including duration of use, methods discontinuation and switching, and dual methods use. Understanding these aspects is important for ongoing FP promotion and also for further interpretation of the preferences and intentions to use FP methods that have been reported in this study. Since our study was based on data collected for another primary purpose, our analysis was restricted to variables that were available in the dataset, and this limited our ability to conduct any further analysis on any other FP aspects of interest. In addition, while we were interested in conducting further stratified analyses on the use of long-term and permanent contraceptive methods, the numbers of those who reported using these methods was very small, limiting any further analysis in this regard. Despite these limitations, our findings have got clear implications for FP promotion in Uganda. We found that while short-term methods continued to be the main methods of preference, there was an indication that preference of long-term methods is generally higher than reported in other studies [5], and indeed, both users and non-users of FP methods indicated intention to use some of these methods. These findings suggest that uptake of long-term contraceptive methods can be improved with more targeted FP promotional campaigns. In addition, the finding that friends and the media are trusted sources of FP methods suggests a need for a more diversified approach to FP promotion that uses alternative mechanisms of FP promotion in addition to the more conventional, clinic-based methods, particularly if we have to reach the 54% of non-users who reported no intention to use FP methods in the next 12 months. The use of peer-to-peer approaches [26] and reproductive health groups have been found to increase FP use among women of reproductive age, suggesting that increased use of these informal mechanisms in addition to more targeted promotions through the mass media can increase uptake of FP services, particularly among non-users.

## Conclusion

Our findings show that knowledge of FP methods was nearly universal and that six in ten women reported currently using a method of FP. We found that clinic providers, friends and the media were the most trusted sources of FP while government and private health facilities were the leading sources of FP services. There was a strong preference for short-term methods although preference of and intention to use long-term methods is higher than among the rest of the Ugandan women. Our findings suggest a need for expanding the spectrum of channels used to disseminate information on contraceptive products and services to include informal channels (such as friends) and the media in order to reach out to potential users of FP methods.

### What is known about this topic

- While knowledge of FP has significantly increased over the last decade; uptake of FP services has remained low, especially in sub-Saharan Africa.
- Lack of knowledge of where to obtain FP methods and lack of information on what women consider to be trusted sources of FP information and services, are key barriers that affect access to and utilization of FP methods in most sub-Saharan African countries.
- Although there has been method-specific increases in the use of short-term methods, uptake of long-term and permanent methods has remained low over the years.

### What this study adds

- Six in every ten women reported currently using a method of family planning; seven in ten of these were using a modern method of family planning. This is higher than what has been reported in other studies.
- Clinic providers, friends and the media are the most trusted sources of contraceptive information while government and private health facilities are the main sources of FP methods.
- Although preference for permanent methods (i.e. female sterilization) remains generally low, preference for and intention to use long-term methods (e.g. implants) is second to injectables among both users and non-users.
